# Spleen nodules: a potential hallmark of Visceral Leishmaniasis in young children

**DOI:** 10.1186/s12879-014-0620-2

**Published:** 2014-12-12

**Authors:** Fraia Melchionda, Stefania Varani, Filomena Carfagnini, Tamara Belotti, Trentina Di Muccio, Roberto Tigani, Rosalba Bergamaschi, Andrea Pession

**Affiliations:** Pediatric Hematology and Oncology Unit St. Orsola-Malpighi Hospital University of Bologna, Via Massarenti 11, Bldg 13, Bologna, 40138 Italy; Unit of Clinical Microbiology, Regional Reference Centre for Microbiological Emergencies (CRREM), St. Orsola-Malpighi University Hospital, Bologna, Italy; Department of Experimental, Diagnostic and Specialty Medicine, University of Bologna, Bologna, Italy; Pediatric Radiology Unit, St. Orsola-Malpighi Hospital University of Bologna, Bologna, Italy; Unit of Vector-Borne Diseases and International Health, MIPI Department, Istituto Superiore di Sanità, Rome, Italy; Pediatric Unit, St. Orsola-Malpighi Hospital University of Bologna, Bologna, Italy

**Keywords:** Visceral leishmaniasis, Hemophagocytic lymphohistiocytosis, Abdominal ultrasonography

## Abstract

**Background:**

Visceral leishmaniasis (VL) is a severe disease caused by *Leishmania infantum* in the Mediterranean basin, and is associated with considerable morbidity and mortality. Infantile VL may begin suddenly, with high fever and vomiting, or insidiously, with irregular daily fever, anorexia, and marked splenomegaly. Delays in diagnosis of VL are common, highlighting the need for increased awareness of clinicians for VL in endemic European countries.

**Case presentation:**

We report 4 cases of young children in northern Italy presenting with persistent fever of unknown origin and diagnosed with VL by serological and molecular methods. At the time of diagnosis, these patients showed an unusual echographic pattern characterized by multiple iso-hypoechoic nodules associated with splenomegaly.

**Conclusion:**

We suggest that detection of spleen nodules represents a signature of VL in infants, thus helping to diagnose systemic *Leishmania infantum* infection in children.

**Electronic supplementary material:**

The online version of this article (doi:10.1186/s12879-014-0620-2) contains supplementary material, which is available to authorized users.

## Background

Visceral leishmaniasis (VL), or kala-azar, is a severe disease caused by various *Leishmania* species belonging to the *Leishmania donovani* complex and is associated with considerable morbidity and mortality [1]. In VL, parasites spread systemically to propagate in the macrophages of internal organs such as the liver, spleen, bone marrow, and lymph nodes. VL is observed in immunocompetent hosts in endemic areas, but it occasionally occurs as complication of immunodeficiency and autoimmune diseases.

The Mediterranean type of VL is usually caused by *Leishmania infantum* [2]. Dogs are the main reservoirs of this parasite that frequently infects infants and young children up to 4 years of age [1]. Since the 1990s, human VL has been on the increase in Italy, with new foci detected not only within endemic areas, but also in northern regions previously regarded as non-endemic [3]. In 2013, a dramatic increase of VL cases has been reported in Bologna Province, which is located in north-eastern Italy [4]. During this outbreak, 5 pediatric cases were also described.

Infantile VL may begin suddenly, with high fever and vomiting, or insidiously, with irregular daily fever, anorexia, weight loss, and pallor. Splenomegaly develops gradually and may become marked; it is evident by the end of the first month of symptom initiation [5].

Here, we report 4 cases of young children presenting with persistent fever of unknown origin (FUO) diagnosed as VL in Bologna Province, Italy. These patients had splenomegaly and an echographic pattern characterized by widespread echostructural alteration of the spleen and the presence of multiple iso-hypoechoic nodules, mostly smaller than 1 cm. Such a pattern has been previously reported only occasionally in association with VL.

## Case presentation

Four children were admitted to the St. Orsola-Malpighi University Hospital (Bologna, Italy) from March to April 2013 because of persistent fever. All patients resided in Bologna Province. In all cases, VL began with unexplained fever, weight loss, and mild-to-moderate anemia.

### Patient 1

**Figure 1:**
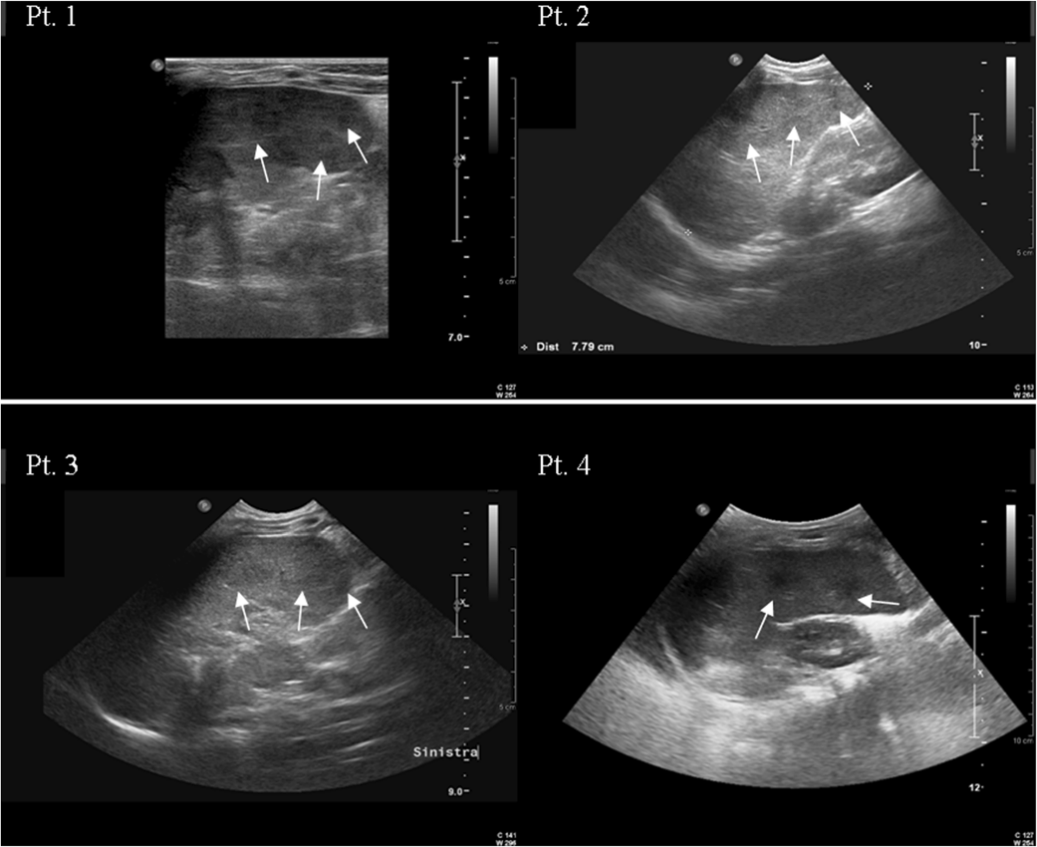
**Spleen sonography of 4 patients showing splenomegaly with multiple small iso-hypoechoic nodules (arrows).**

A 5 month-old girl was admitted to a secondary hospital with a 2-week history of weight loss and fever unresponsive to antibiotic therapy. She was born at term after a physiological pregnancy, and gained weight normally after birth. During this first admission, serological tests for human immunodeficiency virus (HIV), Epstein-Barr virus (EBV), hepatitis A, B, and C viruses, cytomegalovirus (CMV), parvovirus B19, salmonella, and brucella yielded negative results. Aerobic and anaerobic blood cultures, tuberculin sensitivity test, thoracic radiography, and abdominal ultrasonography showed no specific cause for the fever. The patient was transferred to St. Orsola-Malpighi University Hospital for evaluation after 4 weeks of fever and the appearance of moderate three-lineage cytopenias. Serological studies for human herpesvirus-6 (HHV6), measles, adenovirus, *Borrelia* spp., and *Toxoplasma gondii* yielded negative results. Microscopic examination of the bone marrow aspirate showed hemophagocytosis features and absence of *Leishmania* amastigotes. Abdominal ultrasonography revealed a markedly enlarged spleen with diffuse multifocal hypoechoic nodules (Figure 1, Pt 1).

Serum abnormalities included elevated ferritin level (13362 ng/mL) indicating severe inflammation, increased soluble interleukin-2 receptor (sIL2R; >7500 UI/mL), hypertriglyceridemia (674 mg/dL), hypofibrinogenemia (100 mg/dl), severe hypoalbuminemia, anemia (hemoglobin [Hb], 8 g/dL), neutropenia (WBC count, 1650/μL), and thrombocytopenia (platelets [PLT], 100000/μL). Differential diagnosis included familial hemophagocytic lymphohistiocytosis (HLH) syndrome, as 6 of the 8 diagnostic criteria for HLH proposed by the HLH04 study [6] were present in this patient. HLH therapy was initiated, including dexamethasone, cyclosporine, and etoposide, because of the patient’s worsening clinical condition even though no genetic mutations related to familial HLH were present. Serological assays were performed to detect anti-*Leishmania* antibodies; a serum sample of the patient tested positive for anti-*Leishmania* IgG by a rk39-based immunochromatographic test (RapydTest, DID, Italy) [7], and the positivity was confirmed by an indirect immunofluorescence assay (IFAT) performed by the Public Health National reference center (titer: 1:80). A genus-specific real-time PCR for *Leishmania* spp. [8] was then performed on bone marrow aspirate and showed the presence of protozoan DNA, confirming the diagnosis of VL [9]. HLH04 therapy was discontinued, and VL treatment consisting of high-dose liposomal amphotericin B (LAMB) (10 mg/kg IV for 1 day) was carried out. The onset of VL infection was atypical, but consistent with the *Leishmania* transmission period and immaturity of the immune system of this patient. A gradual reduction in ferritin and sIL2R levels occurred after 10–15 days of therapy, and after 1 month, anemia and splenomegaly resolved with reduction of nodules.

### Patient 2

A 1-year-old girl was referred to the St. Orsola-Malpighi University Hospital with a 3-week history of fever and otitis while traveling in Morocco. Laboratory tests showed hyperferritinemia (2330 ng/mL), increased transaminases (aspartate aminotransferase [AST], 172 U/L; alanine aminotransferase [ALT], 185 U/L), hypertriglyceridemia (299 mg/dL), elevated sIL2R (1899 UI/mL), and splenomegaly with multiple iso-hypoechoic nodules (Figure 1, Pt 2). Familial HLH syndrome was considered in the differential diagnosis, but the patient received only 3 of 8 points on HLH scoring. No bacterial or fungal growth was detected by blood culture, and serum samples tested negative for antibodies against herpesviruses, hepatitis viruses, parvovirus B19, adenovirus, HIV, *Borrelia*, and *Toxoplasma*. Microscopic examination of bone marrow showed no *Leishmania* amastigotes. However, the bone marrow sample tested positive for *Leishmania* spp. and for *Leishmania infantum* DNA, respectively by employing a genus-specific [8], and a species-specific real-time PCR [10]. Finally, anti-*Leishmania* antibodies were found in serum by RapydTest and IFAT (titer: 1:160), consistent with a diagnosis of VL. Treatment for VL was carried out with LAMB (10 mg/kg IV), and the fever disappeared after 48 hours; after 2 weeks, decreased levels of inflammatory markers were observed. In addition, reduced splenomegaly and nodules were observed, with complete resolution reached after 1 month and after 4 months, respectively.

### Patient 3

An 18-month-old girl was referred to the St. Orsola-Malpighi University Hospital after 7 days of fever unresponsive to antibiotic therapy. Blood tests showed anemia (Hb, 7.9 g/dl), severe hypertransaminasemia (AST, 1173 U/L; ALT, 1976 U/L), elevated lactated dehydrogenase (LDH, 1066 U/L), hyperferritinemia (555 ng/ml), and hepatosplenomegaly with multiple small iso-hypoechoic splenic nodules (Figure 1, Pt 3). Blood cultures showed no bacterial or fungal growth. Serological tests for herpesviruses, hepatitis viruses, HIV, parvovirus B19, *Borrelia*, and *Toxoplasma*, as well as the tuberculin sensitivity test yielded negative results. No *Leishmania* amastigotes were observed in the bone marrow aspirate, while DNA specific for *Leishmania* spp. and for *L.infantum* was detected by real-time PCR. In addition, RapydTest and IFAT (titer: 1:160) were positive for anti-*Leishmania* antibodies. Treatment for VL with LAMB (10 mg/kg IV) was administered. Defervescence occurred after 4 days, whereas levels of inflammatory and hepatic markers decreased gradually, and Hb level returned to normal within 15 days. Hepatosplenomegaly and nodules completely resolved after 2 months.

### Patient 4

An 18-month-old boy was admitted to the St. Orsola-Malpighi University Hospital with FUO, hypertransaminasemia (AST, 422; ALT, 261 U/L), elevation of inflammatory markers (LDH, 1233 U/L; ferritin, 1750 ng/mL; sIL2R, >7500 UI/mL), and splenomegaly with iso-hypoechoic nodules (Figure 1, Pt 4). At first, the clinical findings were ascribed to EBV infection because of anti-EBV IgM positivity. Nevertheless, the RapydTest was positive for anti-*Leishmania* antibodies, and IFAT was weakly positive (titer, 1:40). No parasites were detected on microscopic examination of a bone marrow smear, while the bone marrow aspirate tested positive for *Leishmania* spp. and for *L.infantum* DNA by real-time PCR. These laboratory findings and the clinical data, including spleen nodularity, suggested a diagnosis of VL, and the patient was treated with LAMB (10 mg/kg IV). The fever resolved within 2 days after treatment. Hepatosplenomegaly and spleen nodules completely resolved after 3 months, inflammatory and hepatic markers decreased gradually to normal levels after 4 months.

Altogether, 4 out of 4 patients were disease-free with a mean follow-up of 18 months (16–20 months).

## Conclusions

Delays in diagnosis of VL are common because of variable incubation time, presence of nonspecific symptoms, difficulty in microscopic identification of rare amastigotes in bone marrow smears, and negative results on serological tests, especially in immunosuppressed patients or in the early stages of HLH caused by *Leishmania* [11]. VL is usually diagnosed by serological tests and parasite detection, the latter including parasitological and molecular methods [12].

In our pediatric cases, microscopic examination of bone marrow smears failed to show the presence of amastigotes. This is not surprising, because the sensitivity of microscopy for detecting VL is 50–80% [12], and this value is further reduced in HLH [11]. Nevertheless, leishmanial DNA was detected in the bone marrow of 4 out of 4 cases, and in 3 out of 4 cases (Patient 2, Patient 3 and Patient 4) we could confirm that VL was caused by *L.infantum*. Thus, in line with published data [9], we observed that molecular methods significantly enhanced *Leishmania* detection in bone marrow samples. In addition, anti-*Leishmania* antibodies were detected in all 4 cases. Thus, molecular and serological tests as well as clinical suspicion were crucial for VL diagnosis in our experience, and positive response to anti-*Leishmania* treatment indicated that our diagnostic approach was effective. All of our patients displayed splenomegaly with peculiar sonographic features of multifocal hypoechoic nodules. We believe that this peculiar ultrasound finding, in association with clinical and laboratoristic criteria, as fever, hepatosplenomegaly, anemia, leucopenia and weight loss, may corroborate the suspicion of VL that should be confirmed by serological and/or molecular tests [13].

Spleen nodules where identified in 4 out of 5 children diagnosed with VL during the recent outbreak occurred in Bologna Province of Italy from November 2012 to May 2013 [4]. However spleen nodules may represent a non specific finding, as they are reported also in several other rare pathologic conditions in children including type I Gaucher disease [14], granulomatous disease, bacterial abscess (staphylococci, streptococci, salmonella, *E.coli*), fungal infection (*Candida albicans*), *Mycobacterium tuberculosis* infection [15], or lymphoproliferative disorders. Case reports of spleen nodules in VL patients have been previously documented by CT scan or MRI [16],[17], however rarely documented in a series of cases as in our report.

VL patients may fulfill several criteria of HLH scoring [6],[18], as in Patient 1; however detection of spleen nodules does not match specifically with familial HLH or HLH secondary to viruses, with only one case reported [19]. Intracellular parasites have an interesting propensity to trigger HLH, and *Leishmania* is one of the most frequently involved non-viral agent. Differential diagnosis between familial and secondary HLH and VL may be difficult, since both pathologic entities may present with nonspecific symptoms and share some laboratory diagnostic features, including marked elevation of inflammatory markers [11],[18],[19].

In conclusion, increasing the number of diagnostic tools to identify VL is crucial to enhance detection of this difficult-to-diagnose infection. In our 4 patients, spleen nodules associated with fever and anemia induced the clinician to further investigate the possibility of VL infection. We suggest that spleen nodules represent a possible hallmark of VL, especially in infants, thus contributing to diagnosis of systemic *L. infantum* infection in children.

## Consent

Written informed consent was obtained from the parents of the patients for publication of this case report and any accompanying images. A copy of the written consent is available for review by the Editor of this journal.

## Authors’ original submitted files for images

Below are the links to the authors’ original submitted files for images. Authors’ original file for figure 1

## Abbreviations

ALT: Alanine aminotransferase
AST: Aspartate aminotransferase
CMV: Cytomegalovirus
EBV: Epstein-Barr virus
FUO: Fever of unknown origin
Hb: Hemoglobin
HHV6: Human herpesvirus-6
HIV: Human immunodeficiency virus
HLH: Hemophagocytic lymphohistiocytosis
IFAT: Indirect immunofluorescence assay
LAMB: Liposomal amphotericin B
LDH: Lactated dehydrogenase
PLT: Platelets
sIL2R: Soluble interleukin-2 receptor
VL: Visceral leishmaniasis
WBC: White blood cell
