# Supplementary figures and images for: Spleen nodules: a potential hallmark of Visceral Leishmaniasis in young children
Source: BMC Infect Dis. 2014 Dec 12;14:620. doi: 10.1186/s12879-014-0620-2 (PMC4270008; doi:10.1186/s12879-014-0620-2)

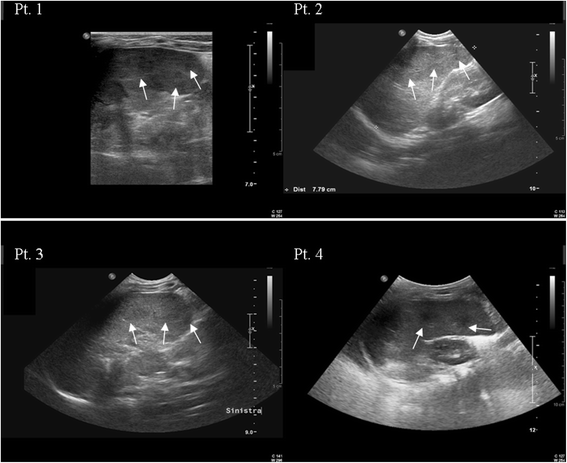

Supplement: Supplementary file 1 — Authors’ original file for figure 1 [file 12879_2014_620_MOESM1_ESM.gif]
